# Genome editing in cells of apple cultivar ‘Fuji’ using geminivirus-derived replicons for transient expression of CRISPR/Cas9 components

**DOI:** 10.5511/plantbiotechnology.24.0903a

**Published:** 2024-12-25

**Authors:** Katsuya Negishi, Masaki Endo, Tomoko Endo, Chikako Nishitani

**Affiliations:** 1 Institute of Fruit Tree and Tea Science, National Agriculture and Food Research Organization, 2-1 Fujimoto, Tsukuba, Ibaraki 305-8605, Japan; 2 Institute of Agrobiological Sciences, National Agriculture and Food Research Organization, 3-1-3 Kannnondai, Tsukuba, Ibaraki 305-8604, Japan

**Keywords:** apple, CRISPR/Cas9, genome editing, *Malus domestica*, replicon

## Abstract

The clustered regularly interspaced short palindromic repeats (CRISPR)/CRISPR-associated protein 9 (Cas9) system has been used for genome editing in various fruit trees, including apple (*Malus* × *domestica*). In previous studies, transfer DNA (T-DNA) expressing genome editing tools, *Streptococcus pyogenes* Cas9 (SpCas9) and single guide RNA (sgRNA), was stably integrated into the apple genome via *Agrobacterium*-mediated transformation. However, due to self-incompatibility, long generation period, and the high heterozygosity of apple, removing only the integrated T-DNA from the apple genome by crossbreeding while maintaining the introduced varietal trait is difficult. Therefore, an efficient SpCas9-sgRNA delivery system without transgene insertion is required for genome editing of apple. In this study, we used geminivirus-derived replicons (GVRs) for the transient expression of genome editing tools. Small DNA vectors were deconstructed by splitting the elements necessary for the production of GVRs from bean yellow dwarf virus into two vectors. Production of GVRs using these vectors was demonstrated in *Arabidopsis* and apple cells. Genome editing was improved by using the GVR-producing vectors with genome editing tools in *Arabidopsis* protoplasts. The use of the GVR-producing vectors for SpCas9 and sgRNA delivery into apple leaves improved the expression levels of SpCas9 and sgRNA, enabling the detection of targeted mutations introduced in the endogenous apple genome. These findings demonstrate the utility of GVRs in genome editing via transient gene expression in apple. It can be expected that our GVR-based genome editing technology has potential utility for transgene-free genome editing in apple.

## Introduction

Apple (*Malus* × *domestica*) is one of the most important and economically valuable fruit trees in the world. In 2022, the global production of apple reached approximately 95.8 million tons, representing a 20% increase over the previous decade (FAOSTAT, http://www.fao.org/faostat (Accessed July 30, 2024)). Although rapid breeding is necessary to address global issues such as climate change and population growth, traditional apple breeding strategies are time-consuming due to challenges such as the long generation period and high heterozygosity of apple (Brown 2012; Visser 1964). The clustered regularly interspaced short palindromic repeat (CRISPR)/CRISPR-associated protein 9 (Cas9) genome editing technology is expected to accelerate plant breeding and is currently being utilized in several fruit tree species (Martín-Valmaseda et al. 2023). CRISPR/Cas9 comprises the Cas9 protein and single guide RNA (sgRNA) complex. The Cas9-sgRNA complex binds to and induces a double-stranded DNA break at target sites complementary to the sgRNA sequence, which is in turn located near the protospacer adjacent motif (PAM) sequence. In the case of the widely used *Streptococcus pyogenes* Cas9 (SpCas9), the length of the target sequence in the sgRNA is 20 nucleotides, and SpCas9 recognizes NGG as a PAM sequence (Jinek et al. 2012; Nishimasu et al. 2014). CRISPR/Cas9 mediated genome editing in apple was first reported in 2016 in the rootstock cultivar ‘JM2’ (Nishitani et al. 2016); since then, several reports of genome editing have been demonstrated in various cultivars (Charrier et al. 2019; Jacobson et al. 2023; Li et al. 2023). In these previous studies, exogenous DNA for SpCas9 and sgRNA expression was introduced into apple cells via *Agrobacterium tumefaciens* as transfer DNA (T-DNA). When T-DNA is stably integrated into the apple genome, SpCas9 and sgRNA are highly expressed and induce targeted mutations efficiently in plant cells. Genome-edited plants can be selected from a population of regenerated plants. In order to generate T-DNA-free null segregants, it is necessary to remove T-DNA from the regenerated plants through the process of crossing. However, due to the high heterozygosity and self-incompatibility of apple, it is difficult to remove only the T-DNA through crossbreeding while maintaining the introduced parental desired trait. *Agrobacterium* is also utilized for the transient expression of transfer genes without nuclear insertion. This enables the generation of T-DNA-free genome-edited plants through the transient expression of genome-editing enzymes using *Agrobacterium* in potato (Umemoto et al. 2023; Yasumoto et al. 2020). In apple, while the efficiency is low, it has been demonstrated that genome editing plants can be generated without T-DNA integration using *Agrobacterium*-mediated transient gene expression (Charrier et al. 2019). However, the number of apple cultivars for which efficient tissue culture, *Agrobacterium*-mediated infection and transformation systems, and other relevant biotechnology techniques have been established remains limited. Therefore, it is important to develop a genome editing strategy for apple trees without using *Agrobacterium* and that does not involve the insertion of T-DNA into the nuclear genome. There are several delivery methods for introducing CRISPR/Cas9 components into plant cells without using *Agrobacterium*, such as polyethylene glycol (PEG) (Woo et al. 2015), particle bombardment (Hamada et al. 2018), and sonication-assisted whisker (Nakamura et al. 2023) methods. These delivery systems enable the introduction of CRISPR/Cas9 components directly into plant cells via DNA vectors, RNA virus vectors, and Cas9-sgRNA ribonucleoprotein (RNP) complexes. Although there has been a report of genome editing in apple protoplasts using RNPs using the PEG method (Malnoy et al. 2016), the number of apple cultivars that can regenerate individuals from protoplasts is also very limited.

Here, we focused on circular geminivirus-derived replicons (GVRs) to establish an efficient DNA vector delivery system to enhance transient gene expression and genome editing. Geminiviruses usually have one or two single-stranded circular DNA molecules of 2,600–2,800 nucleotides (Yang et al. 2017). The replication of geminivirus requires three essential components: a large intergenic region (LIR), a short intergenic region (SIR), and a coding sequence of trans-acting replication initiation protein (Rep)/RepA (Baltes et al. 2014). Rep and RepA proteins are splicing variants and are translated from the same precursor mRNA (Wright et al. 1997). The introduction of DNA fragments containing two LIR and SIR sequences into plants via a DNA vector results in the circulation of GVRs between the LIRs, which replicate autonomously in the presence of Rep (Wang et al. 2017; Yang et al. 2017). DNA vectors using GVRs elements are favored in the development of transient expression technique in plants (Yamamoto et al. 2018). GVRs have been demonstrated to enhance gene expression, thereby increasing the efficiency of CRISPR/Cas9-mediated genome editing (Baltes et al. 2014). In fruit trees, GVRs have been used for genome editing of grapevine (Olivares et al. 2021). In this study, we developed DNA vectors using replicon sequences derived from bean yellow dwarf virus (BeYDV). BeYDV can infect many dicotyledonous plant species (Liu et al. 1997). In addition, it is suggested that GVRs using BeYDV-derived components can replicate in several fruit tree species including apple (Olivares et al. 2021; Omori et al. 2023). In these previous studies, GVR components were loaded onto a single T-DNA construct and introduced into plant cells by *Agrobacterium*. Conversely, it has also been reported that GVR components can be separated into two distinct parts: GVR production and Rep expression parts (Baltes et al. 2014). This vector deconstruction approach would be an effective method for reducing the size of the DNA vector. In this study, the GVR components were separated into two parts and small DNA vectors suitable for DNA transfection were deconstructed. Using these vectors, we demonstrated that GVRs were generated in plant cells from these deconstructed vectors. These GVRs were found to improve the gene expression of SpCas9 and sgRNA. Furthermore, targeted mutations in endogenous sequences in the apple genome were detected using GVRs. These data will provide valuable insight that will facilitate the development and acceleration of genome editing technology in apples in the future.

## Materials and methods

### Vector constructions

The pENTR/D-TOPO cloning vector (Thermo Fisher Scientific, Waltham, MA, USA) was used as a backbone for all vectors used in this study to construct small DNA vectors suitable for plasmid transfection. The sequences inserted into the vectors were amplified from vectors used in the previous studies: LIR, SIR, and Rep/RepA coding sequences from BeYDV (Yamamoto et al. 2018); *Petroselinum crispum* *UBIQUITIN4-2* promoter (PcUBI pro) and *Arabidopsis thaliana* codon-optimized SpCas9 (Mikami et al. 2015); *A. thaliana ALCOHOL DEHYDROGENASE* 5 prime untranslated region (AtADH 5′UTR), simian virus 40 nuclear localization signal (SV40 NLS), *A. thaliana U6-2* small nuclear RNA promoter (AtU6 pro), sgRNA scaffold sequence of SpCas9, and double terminator of cauliflower mosaic virus 35S (35S) terminator and *Agrobacterium tumefaciens* nopaline synthase (nos) terminator (35S-nos ter) (Endo et al. 2019); tomato bushy stunt virus P19 RNA silencing suppressor (p19) (Kaya et al. 2017); and Venus coding sequence (Li et al. 2020). To construct the Rep/RepA expression vector (pRep), LIR, Rep/RepA, and SIR sequences was PCR amplified and cloned into the pENTR vector using an In-Fusion HD Cloning Kit (Takara Bio, Shiga, Japan). To construct the GVR production vector (pGV), LIR and SIR sequences were cloned into the pENTR vector using the In-Fusion HD Cloning Kit. The pGV_reverse-Venus, pGV_SpCas9, and pGV_sgRNA vectors were generated by inserting the expression cassette into the cloning site between LIR and SIR of the pGV vector. The sgRNA expression cassette contains the AtU6 promoter, the 20-nt target sequence, and the sgRNA scaffold sequence. To construct pENTR_Venus, the Venus expression cassette was cloned into the pENTR vector using the In-Fusion HD Cloning Kit. A two-base insertion was introduced into the pENTR_Venus to introduce a frameshift mutation sequence as mutant-Venus and construct pENTR_mutant-Venus.

### Plant material and growth conditions

*Arabidopsis thaliana* L. Columbia-0 line was used in *Arabidopsis* experiments. *Arabidopsis* plants were grown in soil at 22°C under short-day conditions (8 h light/16 h dark). For the apple experiments, *Malus* × *domestica* Borkh cultivar ‘Fuji’ was grown in vitro as described previously (Sato et al. 2023). Apple shoots were cultured at 24°C under long-day conditions (16 h light/8 h dark).

### Protoplast transfection of *Arabidopsis*

*Arabidopsis* leaf protoplasts were prepared and transfected according to a previous study (Endo et al. 2019). Protoplasts were isolated from mesophyll cells in adult leaves of 5-week-old plants by using Tape-Sandwich method (Wu et al. 2009). Plasmid DNA vectors (3 µg for each vector) were introduced into 1×10^5^ protoplasts using the PEG method (Endo et al. 2019). After 2 days of incubation at 22°C in the dark, DNA was extracted using a NucleoSpin Tissue XS kit (MACHEREY-NAGEL, Düren, Germany).

### Particle bombardment for apple leaves

Young leaves measuring less than approximately 2–3 mm from the shoot apex of in vitro-cultured apple plants were excised and placed on Murashige and Skoog Basal medium (Merck, Darmstadt, Germany). The leaf explants were cultivated in the dark for five days and approximately 30 leaf explants utilized per bombardment. Plasmid DNA vectors were coated onto 0.3-µm-diameter gold particles (InBio Gold, Hurstbridge, Australia) by mixing 90 µg of gold particles and 500 µg each of DNA vectors in a solution containing 945 mM CaCl_2_ and 150 mM spermidine. These gold particles were washed with ethanol, dissolved in 5 µl of water, and introduced into apple leaves at 1,300 psi using a PDS-1000/He system (Bio-Rad, Hercules, CA, USA). After 2 days of incubation at 24°C in the dark, the DNA derived from each bombarded apple leaf was extracted into 20 µl of extraction solution using a NucleoSpin Tissue XS kit.

### Fluorescence observation

The excitation and emission peaks of the Venus protein have been observed at wavelengths of 515 nm and 528 nm, respectively (Nagai et al. 2002). For observation of *Arabidopsis* protoplast cells, microscopy was preformed using the Nikon ECLIPSE TS2-FL inverted microscope (Nikon, Tokyo, Japan) and Venus fluorescence was detected through the C-LED470 filter (excitation wavelength: 470/40 nm, dichroic mirror: 500 nm, emission wavelength: 534/55 nm). The fluorescent images were captured using a Nikon DS-L4_DS-Fi3 (Nikon) with an exposure time of 0.1 s and an ISO sensitivity of 1,600. The measurements of number of cells expressing Venus were performed with help of the ImageJ 1.54g (Wayne Rasband and contributors, National Institutes of Health, USA). For observation of apple leaves, microscopy was preformed using the using the Leica M165 FC Fluorescent Stereo Microscope (Leica Microsystems, Wetzlar, Germany) and Venus fluorescence was detected through the GFP-LP filter (LEICA#10447407, excitation wavelength: 460–500 nm, emission wavelength: 510 nm long-pass). The fluorescent images were captured using a Canon EOS Kiss X5 (Canon, Tokyo, Japan) with an exposure time of 0.5 s and an ISO sensitivity of 3,200.

### PCR-based detection of plasmid DNA and circularized GVRs into apple leaves

**Figure 1:**
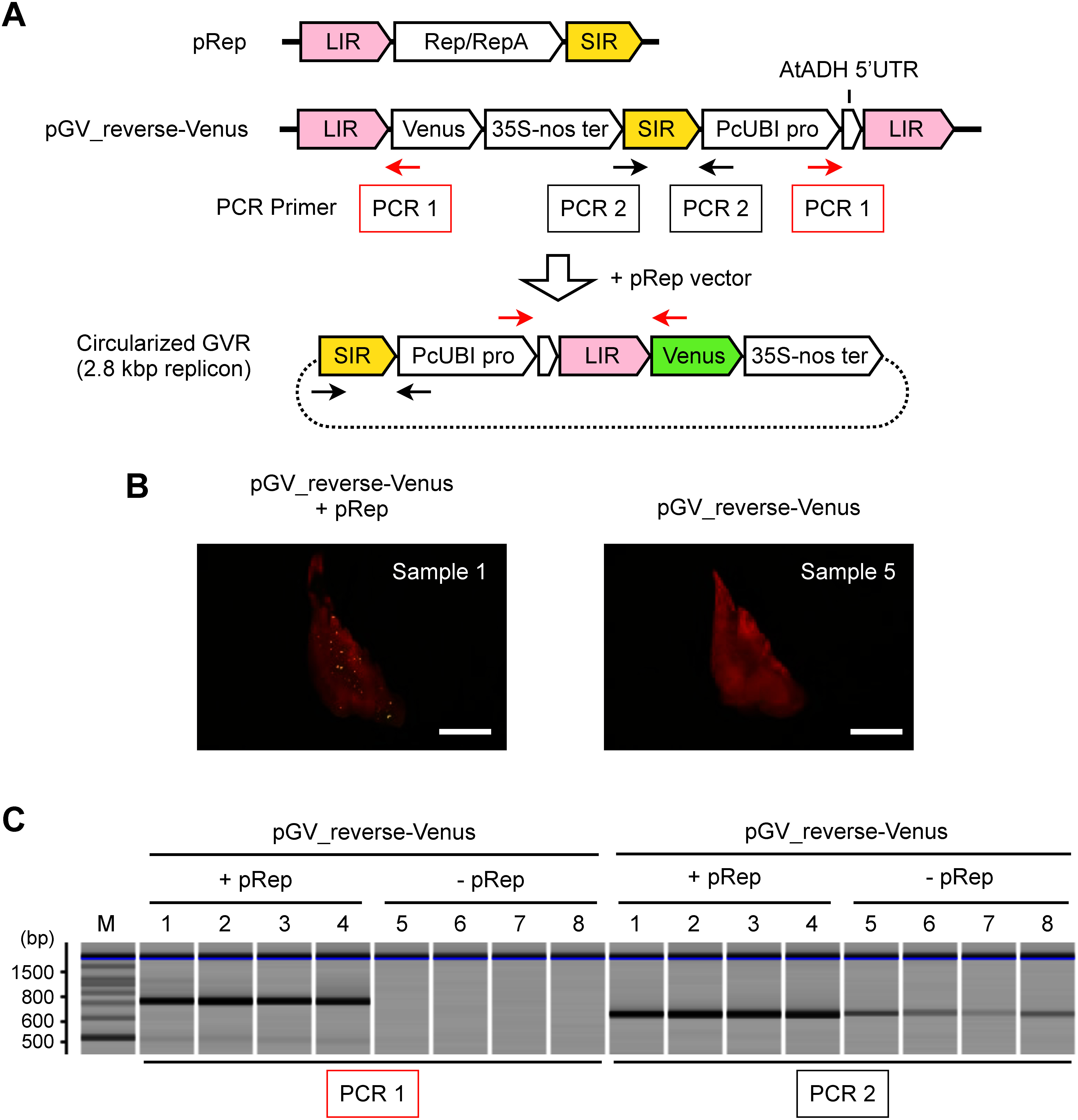
Figure 1. GVR production in plant cells from deconstructed vectors. (A) Structure of pRep, pGV_reverse-Venus vectors, and circularized GVR from pGV_reverse-Venus vector. LIR, large intergenic region; Rep, trans-acting replication initiation protein; SIR, short intergenic region; Venus, Venus coding sequence; 35S-nos ter, double terminator of cauliflower mosaic virus 35S terminator and *Agrobacterium tumefaciens* nopaline synthase terminator; PcUBI pro, *Petroselinum crispum* *UBIQUITIN4-2* promoter; AtADH 5′UTR, *A. thaliana* *ALCOHOL DEHYDROGENASE* 5 prime untranslated region. The direction of each array indicates the sequence direction from the 5′ to the 3′ side. The red arrows (PCR 1) indicate the position of primers for the detection of circularized GVRs. The black arrows (PCR 2) indicate the position of primers in the pGV_reverse-Venus vector. (B) Apple leaves expressing Venus. Sample 1, apple leaf transfected with pGV_reverse-Venus and pRep vectors simultaneously; Sample 5, apple leaf transfected with only pGV_reverse-Venus vector as a negative control. These images were taken through a fluorescent filter. The numbers in the sample correspond to the PCR sample numbers shown in Figure 1C. Bars, 1 mm. (C) PCR-based detection of circularized GVRs within plant cells. M, DNA molecular weight marker. Samples 1–4 (+pRep) were transfected with pGV_reverse-Venus and pRep vectors simultaneously. Samples 5–8 (−pRep) were transfected with pGV_reverse-Venus vector only as a negative control. PCR 1 was used to detect circularized GVRs. PCR 2 was used to indicate the presence of pGV_reverse-Venus vector. Images of samples are shown in Figure 1B and Supplementary Figure S1B.

0.5 µl extracted DNA from each bombarded apple leaf fragment was used as a template for the PCR to determine the presence of DNA sequences derived from pGV_reverse-Venus and circularized GVR (Figure 1C). The PCR was performed using KOD One PCR Master Mix (TOYOBO, Osaka, Japan) and the primers shown in Supplementary Table S1 and in a total reaction volume of 10 µl, under the following reaction conditions: pre-denaturation for 2 min at 94°C, followed by 30 cycles of denaturation for 10 s at 98°C, annealing for 5 s at 55°C, and elongation for 2 s at 68°C. The PCR products were diluted 15-fold with water, and analyzed by a microchip electrophoresis using MCE-202 MultiNA with a DNA-500 kit (SHIMADZU, Kyoto, Japan).

### Cloning of an apple genome editing target sequence

Using apple cultivar ‘Golden Delicious’ doubled-haploid tree GDDH13 v1.1 genome sequences (Daccord et al. 2017) obtained from Phytozome (http://www.phytozome.net/ (Accessed July 30, 2024)) as a reference sequence, we designed cloning primers for cloning the 3′UTR of apple MD05G1312300 and MD11G1251800 genes, which are shown in Supplementary Table S1. Genomic DNA of the ‘Fuji’ cultivar around the target sites were amplified by PCR using KOD One PCR Master Mix with the following condition: pre-denaturation for 2 min at 94°C, followed by 40 cycles of denaturation for 10 s at 98°C, annealing for 5 s at 50°C, and elongation for 5 s at 68°C. The PCR fragments were cloned into pCR-Blunt II TOPO vector (Thermo Fisher Scientific), and sequenced by the Sanger method using an ABI3500xL sequencer (Thermo Fisher Scientific).

### RNA extraction and quantitative reverse transcription PCR

Total RNA, including small RNA, was extracted using an miRNeasy Mini kit (QIAGEN, Hilden, Germany) and MaXtract High Density (QIAGEN) according to a previous study (Negishi et al. 2018). Total RNA was treated with DNase I (QIAGEN). For quantitative reverse transcription PCR (RT-qPCR), 1 µg total RNA was reverse transcribed in a 20 µl reaction mixture using a PrimeScript RT reagent Kit (Takara Bio) with 2.5 µM oligo-dT primer for SpCas9, p19, and Venus, and with 0.1 µM specific oligo primer (Supplementary Table S1) for sgRNA reverse transcription. The resulting cDNA solution was diluted 3-fold with water, and 1 µl was analyzed. RT-qPCR experiments were performed using TB Green Premix Ex Taq II (Takara Bio) with the Applied Biosystems ViiA 7 Real-Time PCR System (Thermo Fisher Scientific). The following program was used for amplification: pre-denaturation for 30 s at 95°C, followed by 40 cycles of denaturation for 5 s at 95°C, annealing for 30 s at 55°C, and elongation for 30 s at 72°C. The specificity of the amplification was verified via melting curve analysis. The relative expression levels of SpCas9, p19, and sgRNA were normalized using Venus expression. Three biological replicates with three technical replicates were used for the quantification. Oligonucleotide primers used for RT-qPCR are shown in Supplementary Table S1.

### Heteroduplex mobility assay

Two days after plasmid DNA transfection by the particle bombardment method, approximately five leaves exhibiting Venus fluorescence indicative of DNA vector transfection were sampled as a single unit for HMA. DNA extraction was conducted using the NucleoSpin Tissue XS kit. The target loci were amplified by PCR using the primers shown in Supplementary Table S1 and KOD One PCR Master Mix with the following condition: pre-denaturation for 2 min at 94°C, followed by 40 cycles of denaturation for 10 s at 98°C, annealing for 5 s at 50°C, and elongation for 5 s at 68°C. The PCR products were diluted 15-fold with water, and analyzed by a microchip electrophoresis using MCE-202 MultiNA with a DNA-500 kit.

### Sequencing analysis

﻿PCR products were cloned into the pCR-Blunt II TOPO vector. The 88 (for MD05G1312300 target sites) and 75 (for MD11G1251800 target sites) *Escherichia coli* colonies harboring pCR-Blunt II-TOPO vector were independently subjected to colony PCR respectively, using KOD One PCR Master Mix with the following condition: pre-denaturation for 2 min at 94°C, followed by 30 cycles of denaturation for 10 s at 98°C, annealing for 5 s at 50°C, and elongation for 5 s at 68°C. The colony PCR products were sequenced by the Sanger method using an ABI3500xL sequencer. The mutation frequency was measured as the ratio of clones with mutation to the total number of sequenced clones.

## Results

### Construction of DNA vectors for development of GVRs in *Arabidopsis* and apple cells

In order to develop small DNA vectors that replicate GVRs in plant cells for transient gene expression, BeYDV-derived GVR components were separated into two DNA vectors: the first (pRep vector) contains LIR, Rep/RepA, and SIR, which expresses Rep and RepA proteins for initiating GVR replication (Figure 1A). The second GVR production vector (pGV vector) contains two LIR sequences and one SIR sequence, which are responsible for generating GVRs. The DNA fragment between the two LIR sequences in the pGV vector becomes a GVR and replicates autonomously in the presence of Rep protein. To investigate GVR production in plant cells from these vectors, we generated a reporter construct, pGV_reverse-Venus vector (Figure 1A). In the pGV_reverse-Venus vector, the absence of a promoter sequence upstream of the Venus coding sequence prevents the expression of Venus in plant cells. On the other hand, the replication-induction by pRep vector will lead to the robust expression of Venus, due to the positioning of the adjacent PcUBI promoter upstream of the Venus sequence in circularized GVRs. We first transfected the pGV_reverse-Venus and pRep vectors into *Arabidopsis* protoplasts using the PEG method (Endo et al. 2019). Transfection of the pGV_reverse-Venus vector alone resulted in only 7% of protoplasts exhibiting Venus fluorescence (Supplementary Figure S1A, left). On the other hand, when the pGV_reverse-Venus vector and the pRep vector were co-transfected simultaneously, strong fluorescence was observed in 58% of total *Arabidopsis* protoplasts (Supplementary Figure S1A, right). This result suggests that GVR production was induced by the pRep vector from the pGV_reverse-Venus vector. To investigate whether these vectors are also effective in apple, we introduced these vectors using the particle bombardment method into excised young leaves of the apple cultivar ‘Fuji’ that were cultured in vitro. The excised apple leaves were transfected with pGV_reverse-Venus and pRep simultaneously (samples 1–4 in Figure 1B, Supplementary Figure S1B) or with pGV_reverse-Venus alone (samples 5–8 in Figure 1B, Supplementary Figure S1B). Two days after transduction, Venus fluorescence was observed, and DNA was extracted for subsequent PCR analysis. Consistent with the results in *Arabidopsis* protoplast, Venus fluorescence was clearly detected only when the pGV_reverse-Venus vector and pRep vector were introduced simultaneously into apple leaves (Figure 1B, Supplementary Figure S1B). PCR analysis showed that PCR products confirming replication were detected only when the pRep vector was introduced simultaneously with the pGV_reverse-Venus vector (Figure 1C). In contrast, PCR products indicating the presence of pGV_reverse-Venus vector were detected in all leaves, suggesting that the pGV_reverse-Venus vector was introduced into all leaves. These results indicate that the deconstructed vectors developed in this study could produce GVRs in *Arabidopsis* and apple cells.

### Improving genome editing efficiency using GVRs in *Arabidopsis* protoplast

**Figure 2:**
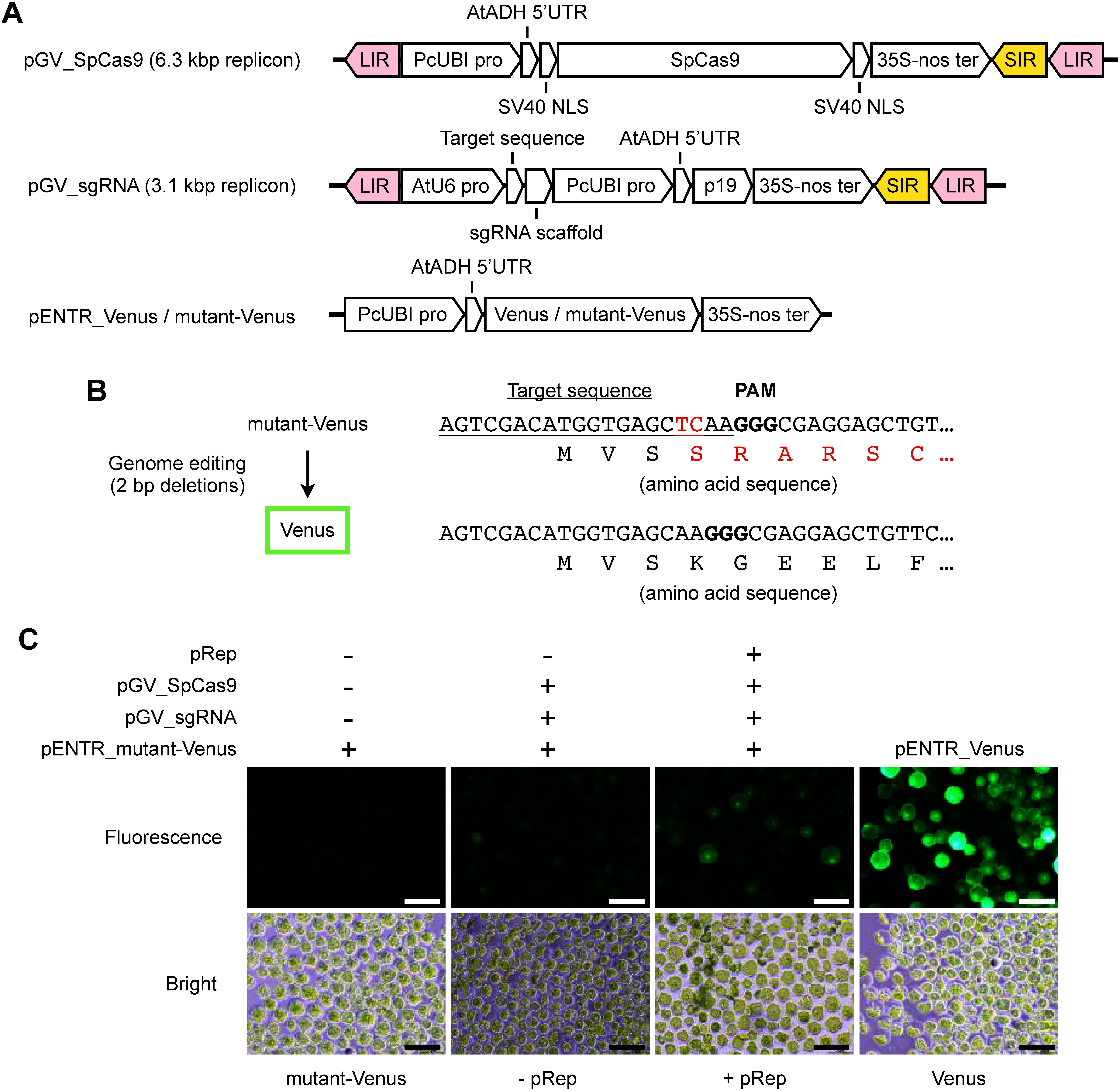
Figure 2. Genome editing for mutant-Venus reporter in *Arabidopsis* protoplasts. (A) Vector constructs of pGV_SpCas9, pGV_sgRNA, pENTR_Venus, and pENTR_mutant-Venus. LIR, large intergenic region; SIR, short intergenic region; Venus, Venus coding sequence; 35S-nos, double terminator of cauliflower mosaic virus 35S terminator and *Agrobacterium tumefaciens* nopaline synthase terminator; PcUBI pro, *Petroselinum crispum* *UBIQUITIN4-2* promoter; AtADH 5′UTR, *A. thaliana* *ALCOHOL DEHYDROGENASE* 5 prime untranslated region; SV40 NLS, simian virus 40 nuclear localization signal; SpCas9, SpCas9 coding sequence; AtU6 pro, *A. thaliana U6-2* small nuclear RNA promoter; sgRNA scaffold, sgRNA scaffold sequence of SpCas9; p19, tomato bushy stunt virus P19 RNA silencing suppressor; mutant-Venus, mutant-Venus coding sequence. The direction of each array indicates the sequence direction from the 5′ to the 3′ side. (B) Target sequence in the pENTR_mutant-Venus. The underlined sequences indicate the 20 bp target sequence of the mutant-Venus sgRNA. Red characters in the nucleotide sequence (TC) indicate the two-base insertion in mutant-Venus. Bold characters (GGG) indicate the PAM sequence. The red characters in the amino acid sequence of mutant-Venus indicate the amino acid mutations due to the two-base insertion. (C) *Arabidopsis* protoplasts following genome editing mutations introduced in mutant-Venus. The upper and lower pictures were generated with the same protoplasts under fluorescent filter (upper) and bright field (lower). The following vectors were introduced in each sample. mutant-Venus, pENTR_mutant-Venus; −pRep, pENTR_mutant-Venus, pGV_SpCas9, and pGV_sgRNA for mutant-Venus; +pRep, pENTR_mutant-Venus, pGV_SpCas9, pGV_sgRNA for mutant-Venus, and pRep; Venus, pENTR_Venus. Bars, 100 µm.

To investigate whether pRep and pGV vectors can be used for genome editing, we developed constructs for SpCas9 and sgRNA expression based on pGV vectors. To be able to visualize genome editing, we also developed a reporter construct containing a Venus gene with a frameshift mutation (mutant-Venus). The size of circular GVRs produced from the pGV_SpCas9 vector is about 6.3 kbp and they contain SpCas9 under the control of the PcUBI promoter (Figure 2A). The pGV_sgRNA for mutant-Venus vector consists of two parts: a sgRNA expression cassette under the control of the AtU6 promoter, and a p19 expression cassette with PcUBI promoter to enhance transient transgene expression (Qiu et al. 2002); the size of GVRs from the pGV_sgRNA for mutant-Venus vector is about 3.1 kbp (Figure 2A). The pENTR_mutant-Venus vector is not code for a functioning Venus due to a frameshift mutation associated with a two-base insertion (Figure 2A, B, Supplementary Figure S2A). If genome editing is introduced and a frameshift occurs at the target site, Venus fluorescence is expected to be recovered. pENTR_Venus (Figure 2A) was used as a positive control to confirm the efficiency of plasmid DNA introduction into protoplasts and the expression of Venus. We introduced these vectors into *Arabidopsis* protoplasts to determine the frequency of cells expressing Venus fluorescence (Figure 2C, Supplementary Figure S2B). In the case of protoplasts in which only the pENTR_mutant-Venus vector was introduced, no Venus fluorescence was observed (Figure 2C: mutant-Venus). When pGV_SpCas9, pGV_sgRNA for mutant-Venus, and pENTR_mutant-Venus vectors were co-introduced (without pRep), Venus fluorescence was detected in 8.5% protoplasts (Figure 2C: −pRep). In contrast, when four DNA vectors (pRep, pGV_SpCas9, pGV_sgRNA for mutant-Venus, and pENTR_mutant-Venus) were introduced simultaneously into *Arabidopsis* protoplasts, Venus fluorescence was observed in 21.1% protoplast cells (Figure 2C: +pRep) These experiments showed that introduction of pRep increases the frequency of fluorescent protoplasts. Mutation patterns were revealed by extracting DNA from these protoplasts 2 days after transfection. The DNA surrounding the target site was amplified and its sequence was analyzed. A two-base deletion was identified in a sample in which pRep was introduced simultaneously, indicating recovery of the in-frame Venus sequence, which was consistent with the fluorescence observation, whereas no in-frame Venus sequence was detected from a sample without pRep (Supplementary Figure S2C, D). These results suggest that using GVRs could enhance genome editing via transient expression of SpCas9 and sgRNA in *Arabidopsis* protoplast.

### GVRs could improve the expression of genome editing components in apple cells

**Figure 3:**
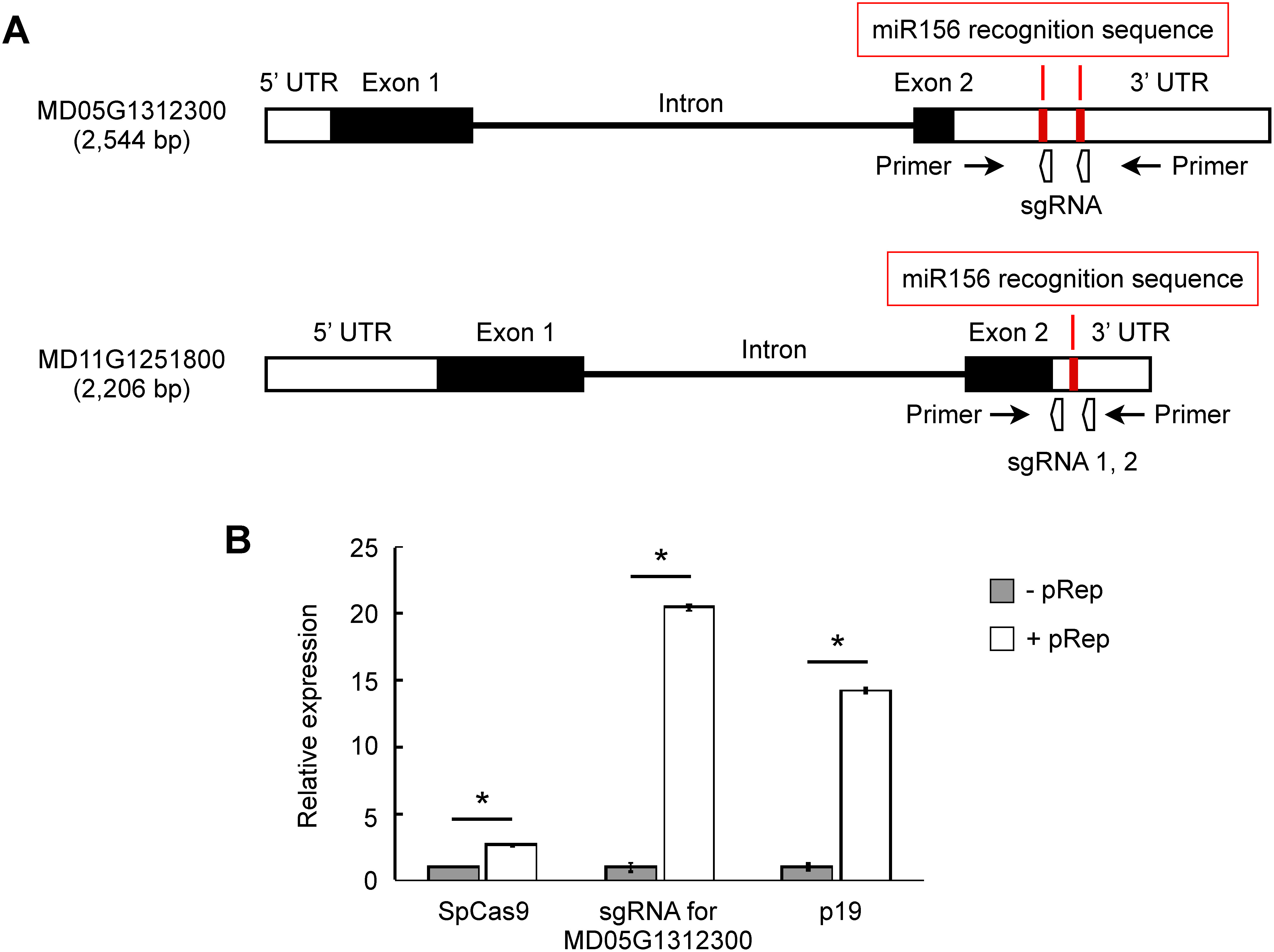
Figure 3. Evaluation of SpCas9 and sgRNA expression using GVR-based transient gene expression. (A) Gene structures of MD05G1312300 and MD11G1251800. The white boxes indicate the UTR, the black boxes represent an exon, and the black bars indicate an intron. The red lines into the 3′UTR indicate the miR156 recognition sequences. The direction of the sgRNAs indicates the sequence direction from the 5′ to the 3′ side. The black arrows indicate the position of primers for cloning and sequencing in Table 1. (B) The relative expression levels of SpCas9, sgRNA for MD05G1312300, and p19 in apple leaves. −pRep, samples transfected with pGV_SpCas9, pGV_sgRNA for MD05G1312300, and pENTR_Venus vectors without pRep vector as a negative control; +pRep, samples transfected with pGV_SpCas9, pGV_sgRNA for MD05G1312300, pENTR_Venus, and pRep vectors simultaneously for GVR production. The presence of asterisks indicates a significant difference at *p*<0.05 between the −pRep and the +pRep (Student’s *t*-test). The data represent means±SE of three biological replicates.

To determine whether the use of GVR-producing vectors is also effective for genome editing in apple, two apple genes (MD05G1312300 and MD11G1251800) were selected as target sites for the introduction of mutations into the endogenous genome of apple. These genes are homologs of *Arabidopsis* *SQUAMOSA PROMOTER BINDING PROTEIN-LIKE3* (*AtSPL3*) which has microRNA156 (miR156) recognition sequence within the 3′UTR (Gandikota et al. 2007; Wang et al. 2009). The 3′UTR sequences of MD05G1312300 and MD11G1251800 were identified from ‘Fuji’ genomic DNA via a process outlined in Materials and methods, and the sgRNAs were designed to delete miR156 recognition sequences in the 3′UTR of these genes. In MD05G1312300, there are two miR156 recognition sequences in the 3′UTR repeat sequence, and one sgRNA was developed to bind to two DNA locations (Figure 3A, Table 1). In contrast, in MD11G1251800, two different sgRNAs were designed to bind upstream and downstream of a single miR156 recognition sequence (Figure 3A, Table 1). It was confirmed that the off-target candidate sequence—defined as a sequence with NGG PAM and a matching seed sequence within 12 PAM-proximal bases, with a maximum of two bases mismatches within a total 20 bases (Nishimasu et al. 2014)—was not presented. The sequence was not identified in the apple cultivar ‘Golden Delicious’ draft genome assembly v1.0 (Velasco et al. 2010) using GGGenome (https://gggenome.dbcls.jp/en/ (Accessed July 30, 2024)), which is an ultrafast nucleotide sequence search engine. Furthermore, no other genomic region was identified as a target using the ‘Fuji’ whole genome v1.0.a2 as a reference sequence (Li et al. 2024) by the genome database for Rosaceae BLAST search tool (https://www.rosaceae.org/blast (Accessed July 30, 2024)). Based on these results, we constructed pGV_sgRNA vectors for MD05G1312300 and MD11G1251800, respectively. For genome editing of MD11G125180, two pGV_sgRNA vectors, each expressing a distinct sgRNA, were constructed and introduced into the apple cells simultaneously. To investigate whether GVR production can improve transient gene expression in apple, SpCas9, sgRNA for MD05G1312300, and p19 expression levels were measured by RT-qPCR. In addition to pRep, pGV_SpCas9, and pGV_sgRNA for MD05G1312300 vectors, pENTR_Venus vector was introduced simultaneously into apple leaves to confirm that the DNA vectors had been transfected by particle bombardment. Leaf fragments containing DNA-transfected cells were sampled using Venus fluorescence as a marker, and the relative expression levels were normalized using Venus expression as a control. The primers used in RT-qPCR are shown in Supplementary Table S1. RT-qPCR analysis demonstrated that expression levels of the transfected genes on GVR production vectors were upregulated significantly (2.6-fold for SpCas9, 20.4-fold for sgRNA, and 14.2-fold for p19) in apple cells when the pRep vector was introduced simultaneously, compared with when it was not introduced (Figure 3B). These results indicate that the expression of genome editing tools can be upregulated in apple cells through the use of GVRs.

**Table 1:** Table 1. The nucleotide sequences around target sites in apple genome.

Target gene	Sequence
MD05G1312300	GAAAACATTTTAAGTGCTTTTAGAACCAAAAAATATTTTCTTTAAAAGTACTTTTAACCATTTTAAAAACACATCCAAACGAGCTCTTATTAGTTTCCATCTAGCATACGATAAGCT*CCC*TATCTTCTGTCAACCCTCGGCCTTCCCCCCCCCCAGTGTGCATTAATTAAGGAAGATTGACTTACTTTTAACCATTTTAAAAACACATTCAAACGAGCCCTTATTAGTTTCTACTAAGCATACGATAAGCT*CCC*TATCTTCTGTCAACCCTCGGCCTTCATTATACGTATGCACCACTGAAACTCCCACACCCCACCCCCCGGGCCCCCCGGTATGCATTAATTAAGGAATATTGGCTTCTTTGCTAGCACAATTTTGA
MD11G1251800	GCGAACCTTATGGAGAAAGCTCAAGCCGGAGAGTGGTTGGTCACCAATACAAAGAAAGTCAGACTAGATATCAGATCACGCCCCCAGGAAACTCTTCCTCCAAGCATTGTCAGATCAGATAAGG*CCA*TGCATCTTGTGATCTGCTCTCTACATGCTCTCTCTCTTCTGTCAT*CCG*TGGCTTTGTTTCTAGGCCATTACATATGTGAGCTTCTAAAGCTATTCTGGTGTATGGTTATGTAGTTTCGACTTGTGTTAAAACTAATCCTTAACACTATTCTGGATGTTTCAATAAGACAATTGGGAATG

The red characters indicate the 20 bp target sequences of SpCas9. The italicized characters indicate PAM sequences. The underlined characters indicate the predicted miR156 target sequences.

### Targeted mutagenesis in apple genome using GVRs-producing vectors

We investigated targeted mutations at target sites in the apple genome. As in the experiment that examined gene expression, plasmid DNA vectors were introduced into apple leaves using the particle bombardment method. Genomic DNA was extracted from apple leaves 2 days after the introduction of DNA vectors using Venus fluorescence as a marker. The genome editing target loci were amplified by PCR using the primers shown in Supplementary Table S1, and the resulting PCR products were then analyzed by heteroduplex mobility assay (HMA). In this assay, the presence of mutations within the target sites is indicated by a shift of heteroduplex bands from the homoduplex PCR products via microchip electrophoresis (Endo et al. 2019). In both the MD05G1312300 and MD11G1251800 target sites, no HMA-positive samples were observed in the absence of pRep vector. Conversely, HMA-positive samples could be detected upon transfection of pRep vector (Supplementary Figure S3). PCR products derived from the HMA-positive sample were cloned into the pCR-Blunt II TOPO vector to confirm the mutation patterns (Table 2). As shown in Table 2, we could detect mutations in the apple genome at both target sites of MD05G1312300 and MD11G1251800. In case of MD05G1312300 target site, single sgRNA targets two miR156 recognition sequence. The mutation frequency in the region containing the 5′ miR156 recognition sequence site was higher than that of mutations containing the 3′ miR156 recognition sequence site. In the MD11G1251800 target site, two pGV_sgRNA vectors targeting upstream and downstream of a miR156 recognition sequence were co-transfected. A deletion-type mutation was also detected, although at a low frequency and this mutation was observed in only one of the two target sites of the sgRNA. These results suggest that genome editing frequency by transient gene expression using GVRs is affected by the target sequence and/or its surrounding sequences. Overall, these findings demonstrate the utility of GVRs for genome editing in apple through DNA vector transfection and transient gene expression.

**Table 2:** Table 2. Targeted mutation patterns in apple genome.

Target gene	Sequence	Mutation pattern	No. clone	Frequency (%)
MD05G1312300	AAGCT*CCC*TATCTTCTGTCAACCCTCGGCCTTCCCCCCCCCCAGTGTGCATTAATTAAGGAAGATTGACTTACTTTTAACCATTTTAAAAACACATTCAAACGAGCCCTTATTAGTTTCTACTAAGCATACGATAAGCT*CCC*TATCTTCTGTCAACCCTCGGCCT	WT	60	68.2
	-------ATTCAAACGAGCCCTTATTAGTTTCTACTAAGCATACGATAAGCT*CCC*TATCTTCTGTCAACCCTCGGCCT	−134 bp	22	25.0
	AAGCT*CCC*TAT------CTTCTGTCAACCCTCGGCCT	−134 bp	3	3.4
	------CAAACGAGCCCTTATTAGTTTCTACTAAGCATACGATAAGCT*CCC*TATCTTCTGTCAACCCTCGGCCT	−134 bp	1	1.1
	------CTTATTAGTTTCTACTAAGCATACGATAAGCT*CCC*TATCTTCTGTCAACCCTCGGCCT	−134 bp	1	1.1
	AAGCT*CCC*TATCTTCTGTC(+134bp)AACCCTCGGCCTTCCCCCCCCCCAGTGTGCATTAATTAAGGAAGATTGACTTACTTTTAACCATTTTAAAAACACATTCAAACGAGCCCTTATTAGTTTCTACTAAGCATACGATAAGCT*CCC*TATCTTCTGTCAACCCTCGGCCT	+134 bp	1	1.1
MD11G1251800	GG*CCA*TGCATCTTGTGATCTGCTCTCTACATGCTCTCTCTCTTCTGTCAT*CCG*TGGCTTTGTTTCTAGGCCATTAC	WT	74	98.7
	GG*CCA*TGCATCTTGTGATCTGCTCTCTACATGCTCTCT------	−113 bp	1	1.3

The red characters indicate the 20 bp target sequences of SpCas9. The italicized characters indicate PAM sequences. The underlined characters indicate the predicted miR156 target sequences. The mutation frequencies were measured as the ratio of clones with mutation to the total number of sequenced clones.

## Discussion

In this study, we deconstructed GVRs producing DNA vectors for delivery and transient expression of genome editing tools. As demonstrated by the reverse-Venus reporter construct, the circularized GVR production was induced by using pGV and pRep vectors in *Arabidopsis* and apple cells (Figure 1). The introduction of the pGV_reverse-Venus vector into *Arabidopsis* protoplasts indicated the presence of a limited number of cells exhibiting Venus fluorescence, even in the absence of the pRep vector (Supplementary Figure S1A, left). On the other hand, the PCR analysis in apple suggests that circularized GVR was only produced when the pRep vector was introduced simultaneously (Figure 1C). Therefore, weak Venus expression detected in *Arabidopsis* protoplasts was probably due to the bidirectional promoter activity of the LIR (Hefferon et al. 2006).

The total DNA vector length of the SpCas9, sgRNA, and the essential components for GVR production (LIR, SIR, and Rep/RepA), is usually over 9 kbp including regulatory elements such as promoters and terminators, which is considerably larger than the original BeYDV size (2,561 nucleotides) (Chen et al. 2011). It has been reported that the cargo capacity is limited in both plant RNA virus vector and geminiviral DNA vector, and that the stability of the vector is correlated negatively with the carrying DNA size (Avesani et al. 2007; Suárez-López and Gutiérrez 1997). In the replicons derived from wheat dwarf virus used in rice, transient gene expression is reduced significantly when the insertion DNA fragments in the replicon expression exceeds 5 kbp (Wang et al. 2017). In addition, the large DNA vector size may also result in fragmentation during particle bombardment experiments, which could lead to reduced expression and the undesired insertion of DNA fragments into the plant genome. Therefore, the small GVR production vectors developed in this study will allow us to create small vectors that are suitable for DNA delivery and transient gene expression and preventing unwanted transformation. We developed GVRs producing DNA vectors utilizing the pENTR vector as a backbone, and loaded Rep/RepA, SpCas9, and sgRNA into distinct vectors. The expected replicon sizes of the GVRs containing the SpCas9 and sgRNA expression cassettes in this study are 6.3 kbp and 3.1 kbp, respectively (Figure 2A). RT-qPCR experiments showed that the effect of increased gene expression by GVRs was higher for sgRNA (20.4-fold) and p19 (14.2-fold) compared with SpCas9 (2.6-fold) (Figure 3B). Consistent with previous reports (Wang et al. 2017), our results also suggest that the efficacy of GVRs in enhancing gene expression is dependent on their size, and the DNA length only in the SpCas9 expression cassette may be still larger than the cargo capacity of BeYDV-based GVRs. Consequently, smaller expression systems for genome editing tools will be more effective in gene expression by increasing the number of GVRs within plant cells. For example, a split-Cas9 system, which allows for the division of the Cas9 construct into two parts and the reduction of the vector size, has the potential to enhance GVR production. Kaya and colleagues have demonstrated that the *Staphylococcus aureus* Cas9 (SaCas9; 1,053 amino acids) derived split-SaCas9 system has the potential to reduce the size of the construct that allows for its loading onto a virus vector for use in genome editing in tobacco (Kaya et al. 2017). In addition, it has recently been reported that several smaller Cas variants than SpCas9 (1,386 amino acids), including Cas12j (700–800 amino acids) and SpCas12f (497 amino acids), have been used for genome editing in plants (Bigelyte et al. 2021; Pausch et al. 2020; Sukegawa et al. 2023). These miniature Cas variants can be loaded into GVRs, although the resulting genome editing efficiency is very low due to the low activity of these Cas variants (Gong et al. 2024). The establishment and improvement of a transient gene expression system using GVRs and these Cas variants in future studies may facilitate the efficient introduction of genome editing tools in apple.

We demonstrated targeted mutagenesis in apple genome using GVRs, but genome editing efficiency depended on the target site, and many deletion type mutations exceeding 100 bp were detected (Table 2). The large-scale deletions exceeding 100 bp observed in MD05G1312300 target site may be attributed to the DNA repair process known as single-strand annealing, which is a DNA repair system that occurs between homologous sequences located on both sides of the double-stranded DNA break site (Vu et al. 2022). To generate more diverse mutations, it is necessary to enhance the efficiency of the genome editing. The use of appropriate promoters and codon optimization have also been reported as effective methods to improve genome editing efficiency (Mikami et al. 2015). We used the AtU6 promoter for sgRNA expression and the PcUBI promoter for *Arabidopsis* codon optimized SpCas9 expression (Figure 2A), which are used widely in genome editing in dicotyledonous plants, including in apple (Charrier et al. 2019; Nishitani et al. 2016). In contrast, it has been reported recently that genome editing efficiency in fruit trees such as grapevine is improved by using the plant species’ own U6 promoter rather than the AtU6 promoter (Ren et al. 2021). In apple, MdU6 has been identified and used for genome editing (Charrier et al. 2019). The combination of these technologies with GVR production vectors will be expected to lead to more efficient genome editing technologies.

In this study, plasmid DNA vectors expressing CRISPR/Cas9 components were coated with gold particles and bombarded into apple cells. The generation of transgene-free genome-edited plants using the particle bombardment method has been demonstrated in several previous studies (Hamada et al. 2018; Hoengenaert et al. 2023; Imai et al. 2020). In the case of the poplar tree, plasmid DNAs were introduced into poplar callus, resulting in the formation of 22.9% gene-edited cell clusters that were potentially transgene-free (Hoengenaert et al. 2023). In our experiments, the fluorescence derived from Venus was brightest after two days after particle bombardment and had disappeared by one week in apple cells. This implies that stable integration of plasmid DNA into the genome rarely occurs. However, it is still possible that small DNA fragments from the plasmid vectors may be inserted into the nuclear genome. To confirm the efficacy of the GVRs-producing DNA vector for obtaining transgene-free genome-edited apples, further studies are required to improve the genome editing efficiency and to generate a regenerated genome-edited apple line that demonstrates the absence of DNA insertion. The method of shoot regeneration from leaf segments has been established in several apple cultivars, including ‘Fuji’ (Nishitani et al. 2016; Sato et al. 2023). Nevertheless, the probability of obtaining regenerated individuals derived from genome-edited cells without drug selection or other procedures is anticipated to be relatively low (Charrier et al. 2019). It may be viable to accelerate the redifferentiation of genome-edited individuals through the integration of additional techniques. For example, the use of regeneration factors (Umemoto et al. 2023) or the transient expression of drug resistance genes simultaneously (Nakamura et al. 2023) could potentially enhance the efficiency of this process. In the apple, several genes that promote redifferentiation have been identified (Chen et al. 2022; Dong et al. 2022). The use of these genes with the GVR production vectors will facilitate the development of more efficient technologies for the generation of genome-edited apples in the future.

Recent technological advances in CRISPR/Cas9, including mutagenesis using Cas9 variants with less restrictive PAM sequences (Endo et al. 2019), base editors that introduce base substitutions (Gaudelli et al. 2017; Nishida et al. 2016), and prime editors that utilize RNA templates for introducing precise mutation (Anzalone et al. 2019), have allowed more precise genome editing. These base editors and prime editors are constituted by the fusion of Cas9 variants and DNA deaminase or mutated reverse transcriptase. In previous studies examining the transfection of SpCas9-sgRNA RNP complexes via particle bombardment, the SpCas9 protein, expressed in large quantities in *E. coli*, is purified and utilized (Kumagai et al. 2022). Nevertheless, it is difficult to express the necessary amount of purified base editor for RNP transduction in *E. coli*, and complex experimental systems, such as those employing human cells, are required (Jang et al. 2021). Therefore, the delivery of genome editing tools using DNA vectors represents an effective method for precision genome editing. The development of these genome editing tools using GVRs in apple could therefore facilitate accelerated breeding. For example, in apple, a single nucleotide substitution in *PHYLLO* causes a pale green lethal disorder phenotype (Orcheski et al. 2015), and this mutation is linked to a no-skin-splitting phenotype, which is an important agronomic trait (Kunihisa et al. 2019). Therefore, precise genome editing using GVRs in future studies will facilitate the development of valuable breeding material.

## Abbreviations

ADH: ALCOHOL DEHYDROGENASE 5
BeYDV: bean yellow dwarf virus
Cas9: CRISPR-associated protein 9
CRISPR: clustered regularly interspaced short palindromic repeats
GVR: geminivirus-derived replicon
HMA: heteroduplex mobility assay
LIR: large intergenic region
miR156: microRNA156
nos: nopaline synthase
PAM: protospacer adjacent motif
PEG: polyethylene glycol
p19: tomato bushy stunt virus P19 RNA silencing suppressor
Rep: trans-acting replication initiation protein
RNP: ribonucleoprotein
RT-qPCR: quantitative reverse transcription PCR
sgRNA: single guide RNA
SIR: short intergenic region
SPL: SQUAMOSA PROMOTER BINDING PROTEIN-LIKE
SV40 NLS: simian virus 40 nuclear localization signal
T-DNA: transfer DNA
35S: cauliflower mosaic virus 35S
UBI: UBIQUITIN4-2
U6: U6-2 small nuclear RNA
UTR: untranslated region

## Description of Supplementary Files

Supplementary Figure S1. GVR-mediated Venus expression in *Arabidopsis* protoplasts and apple leaves.

Supplementary Figure S2. Targeted mutagenesis in mutant-Venus.

Supplementary Figure S3. HMA of apple leaves using GVR vectors at MD05G1312300 and MD11G1251800 target sites.

Supplementary Table S1. Primer sequences used for this study.
