# Supplementary material for: Genome editing in cells of apple cultivar ‘Fuji’ using geminivirus-derived replicons for transient expression of CRISPR/Cas9 components
Source: Plant Biotechnol (Tokyo). 2024 Dec 25;41(4):425–36. doi: 10.5511/plantbiotechnology.24.0903a (PMC11897727; doi:10.5511/plantbiotechnology.24.0903a)
Supplement: Supplementary Data [file plantbiotechnology-41-4-24.0903a-s001.pdf]

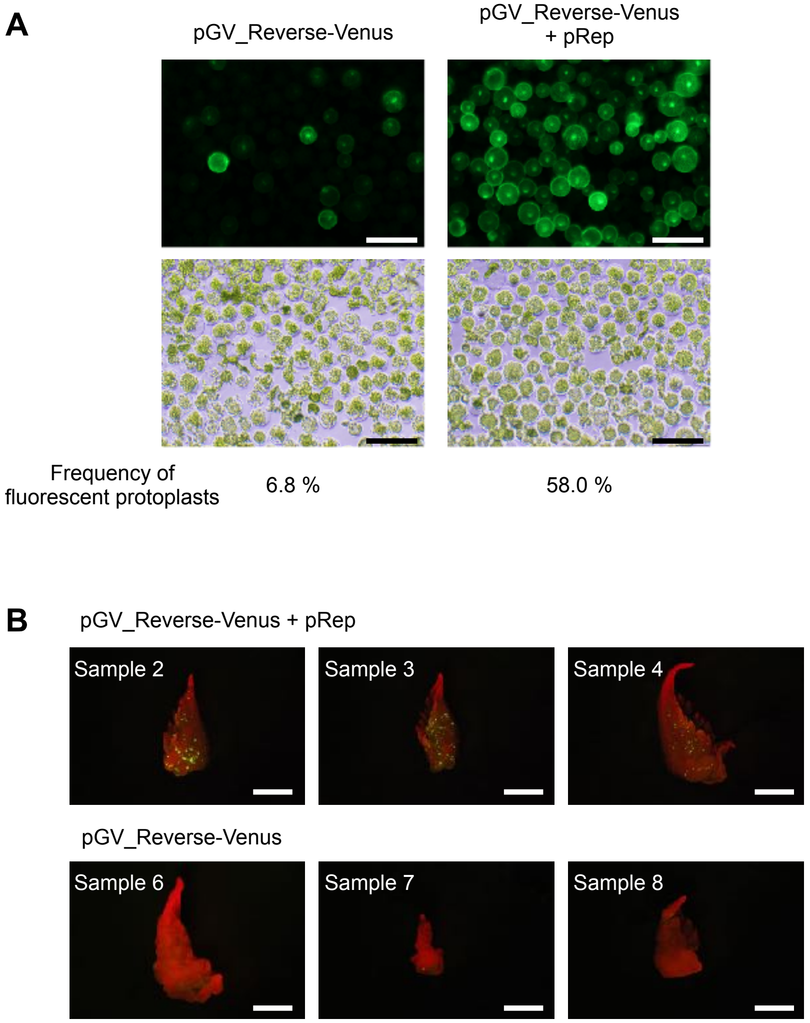

Supplementary Figure S1. GVR-mediated Venus expression in *Arabidopsis* protoplasts and apple leaves.

(A) *Arabidopsis* protoplasts expressing Venus. The upper and lower pictures were generated with the same protoplasts under fluorescent filter (upper) and bright field (lower). Bar, 100 μm.

(B) Apple leaves expressing Venus. These images were taken through a fluorescent filter. The sample 2~4 show apple leaves transfected with the pGV_reverse-Venus and pRep vectors simultaneously, while the sample 6~8 show plants with only pGV_reverse-Venus vector. The numbers in the sample correspond to the PCR sample numbers shown in Figure 1C. Bar,1 mm.

A

Mutant-Venus MVSSRARSCSPGWCPSWSSWTAT*

Venus MVSKGEELFTGVVPILVELDGDVNGHKFSVSGEGEGDATYGKLTLKLICTTGKLPVPWPTLVTTLGY
GLQCFARYPDHMKQHDFFKSAMPEGYVQERTIFFKDDGNYKTRAEVKFEGDTLVNRIELKGIDFKE
DGNILGHKLEYNYNSHNVYITADKQKNGIKANFKIRHNIEDGGVQLADHYQQNTPIGDGPVLLPDNH
YLSYQSALSKDPNEKRDHMVLLEFVTAAGITLGMDELYK*

B

	Vector	Total number	Weak fluorescent	Strong fluorescent
mutant-Venus	pENTR_mutant-Venus	231	0	0
- pRep	pENTR_mutant-Venus<br>pGV_SpCas9<br>pGV_sgRNA	423	30	6
+ pRep	pRep<br>pENTR_mutant-Venus<br>pGV_SpCas9<br>pGV_sgRNA	625	86	46
Venus	pENTR_Venus	373	19	200

C

pRep	Sequence	Mutation pattern	Sequenced clone number	Frequency (%)
-	AGTCGACATGGTGAGCTCAA GGG	mutant Venus	18	100
+	AGTCGACATGGTGAGCTCAA GGG	mutant Venus	14	87.5
	AGTCGACATGGTGAG--CAA GGG	in flame (-2bp)	2	12.5

D

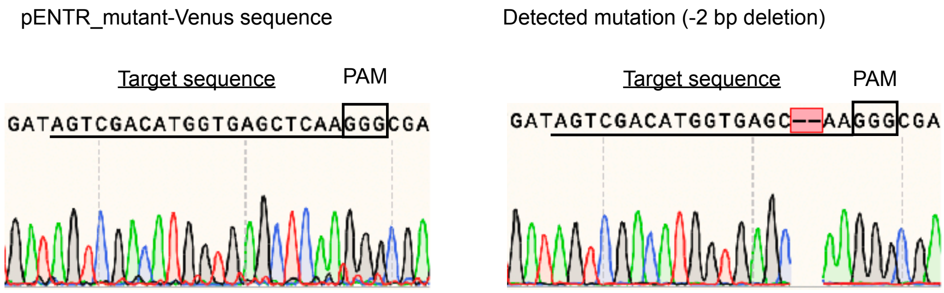

Supplementary Figure S2. The targeted mutagenesis in mutant-Venus.

(A)The amino acid sequence of mutant-Venus and Venus. The red characters indicate the altered amino acid sequence due to two bases insertion in mutant-Venus.

(B)The number of protoplasts of the *Arabidopsis* which were used for the quantification of the frequency of fluorescent protoplast.

(C)The genome editing pattern and frequency of mutant-Venus in *Arabidopsis* protoplasts.

(D)Sanger sequencing chromatograms of mutant-Venus and detected mutants.

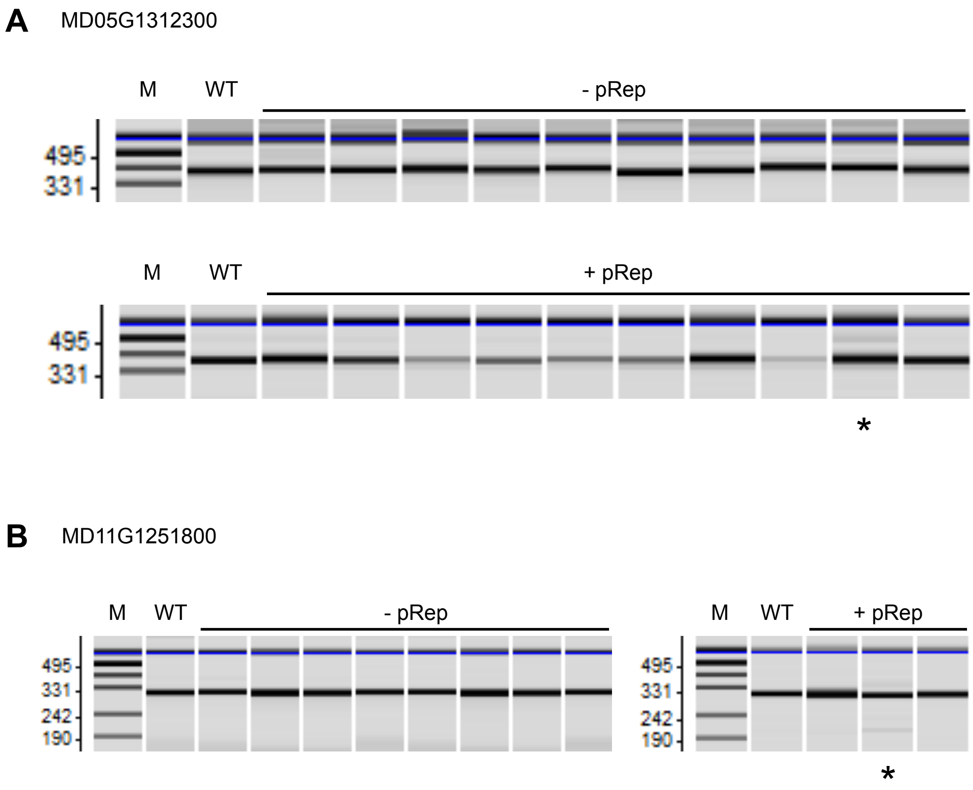

Supplementary Figure S3. HMA of apple leaves using GVR production vectors at MD05G1312300 (A) and MD11G1251800 (B) target sites. M, DNA molecular weight marker; WT, wild-type.

- pRep indicates the sample which were transfected pGV_SpCas9, pGV_sgRNA, and pENTR_Venus vectors without pRep vector as a negative control. +pRep indicates the sample which were transfected with pGV_SpCas9, pGV_sgRNA, pENTR_Venus, and pRep vectors simultaneously for GVRs production. Asterisk, HMA-positive sample.

Supplementary Table S1. Primer list used in this study.

Primer name	Sequence
Venus_PCR 1_Fw	GCATAGATCTGGATTACATGATTGTG
Venus_PCR 1_Rv	AGATCAGCTTCAGGGTCAGCTTGC
Venus_PCR 2_Fw	GGCCGTTTGTTAACGCTGATGTTG
Venus_PCR 2_Rv	CAAAATCCAGTACTAAAATCCAGATCC
mutant-Venus_Fw	GTAGATCTGGACTTTTTGGAGTTGTTGACTTG
mutant-Venus_Rv	AGATCAGCTTCAGGGTCAGCTTGC
sgRNA_RT_specific_oligo	AAAAGCACCGACTCGG
Venus_qPCR_Fw	CCGACAACCACTACCTGAGCTACC
Venus_qPCR_Rv	CTTACTTGTACAGCTCGTCCATGC
SpCas9_qPCR_Fw	TTACCAACCTCGGTGCTCCTGC
SpCas9_qPCR_Rv	CACCGAGCTGTGAGAGATCGATCC
sgRNA_qPCR_Fw	GTTTTAGAGCTAGAAATAGCAAG
sgRNA_qPCR_Rv	CACCGACTCGGTGCCAC
p19_qPCR_Fw	CATCACCGTTTCTGGTGGATCTAGG
p19_qPCR_Rv	ACTCAACCTCGATAGGAGCCAGC
MD05G1312300_Fw	GAAAACATTTTAAGTGCTTTTAGAACC
MD05G1312300_Rv	TCAAAATTGTGCTAGCAAAGAAGCC
MD11G1251800_Fw	GCGAACCTTATGGAGAAAGCTCA
MD11G1251800_Rv	CATTCCCAATTGTCTTATTGAAACATCC
